# A 0.18 μm CMOS LDO Regulator for an On-Chip Sensor Array Impedance Measurement System

**DOI:** 10.3390/s18051405

**Published:** 2018-05-02

**Authors:** Jorge Pérez-Bailón, Alejandro Márquez, Belén Calvo, Nicolás Medrano

**Affiliations:** Group of Electronic Design, Aragon Institute for Engineering Research, I3A, University of Zaragoza, 50009 Zaragoza, Spain; amarquez@unizar.es (A.M.); becalvo@unizar.es (B.C.); nmedrano@unizar.es (N.M.)

**Keywords:** CMOS analog integrated circuits, low dropout regulator (LDO), impedance spectroscopy, sensor array

## Abstract

This paper presents a fully integrated 0.18 μm CMOS Low-Dropout (LDO) Voltage Regulator specifically designed to meet the stringent requirements of a battery-operated impedance spectrometry multichannel CMOS micro-instrument. The proposed LDO provides a regulated 1.8 V voltage from a 3.6 V to 1.94 V battery voltage over a −40 °C to 100 °C temperature range, with a compact topology (<0.10 mm^2^ area) and a constant quiescent current of only 7.45 μA with 99.985% current efficiency, achieving remarkable state-of-art Figures of Merit (FoMs) for the regulating–transient performance. Experimental measurements validate its suitability for the target application, paving the way towards the future achievement of a truly portable System on Chip (SoC) platform for impedance sensors.

## 1. Introduction

Many emerging sensor technologies, especially those based on bio and nano-materials, rely on Impedance Spectroscopy (IS) to evaluate their activity, i.e., the sensor information is obtained from its impedance extraction over a specific interval of stimulus frequencies [1,2,3]. However, despite the versatility and the promising applications of the newest impedance sensors—from environmental monitoring [4] to molecular diagnosis [5,6,7] or DNA or proteins microarrays [8,9]—their potential use outside the specialized laboratories is hindered by the lack of suitable on-chip electronic interfaces that allows preserving levels of resolution, accuracy and reliability comparable to those of the bulky laboratory instruments, but with a miniaturized system powered by limited energy sources. In addition, the trend towards the integration of sensor arrays to permit multi-parameter sensor fusion and improve measurement accuracy imposes even more demanding design restrictions on the electronic circuits of the IS interface.

Typically, these sensor output signals present a low signal-to-noise ratio (SNR), making necessary the use of special techniques for the extraction of the information [9,10,11]. One appropriate low-voltage low-power (LVLP) compatible measurement solution is the use of the Frequency Response Analysis (FRA) or lock-in amplifier-based (LIA) technique that allows using basically two quadrature phase mixers to extract the magnitude and phase information of very small sensor signals at a reference frequency f_0_ even in noisy environments [12]. In addition, one of the key aspects to achieve a truly portable sensing device is the implementation of a System-on-Chip (SoC) solution, integrating in a single die the actuation system, the read-out electronics and an efficient power management unit. This involves CMOS fabrication: with the actual nanometric CMOS technologies, the cost is reduced, the level of integration is noticeably increased, and the integrity of the output signal is maintained, achieving in this way more robust, compact and cheaper solutions.

**Figure 1:**
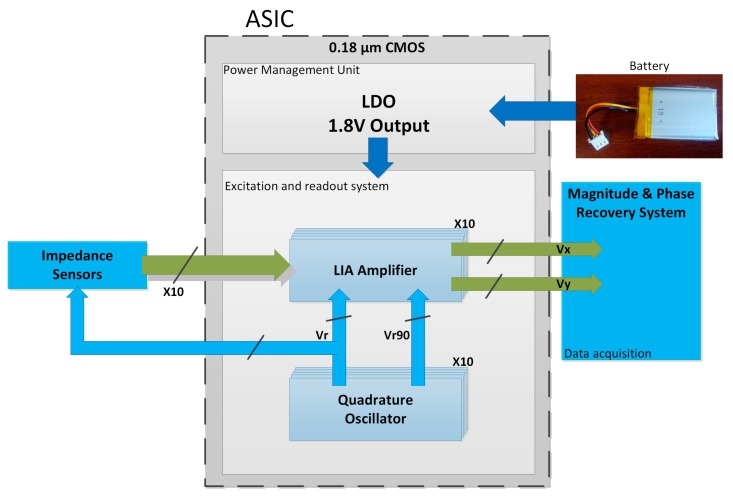
Multichannel Lock-In Amplifier-based (LIA) System on Chip (SoC) portable instrument.

Therefore, to extend the use of miniaturized sensor arrays to applications in which the portability and the ease of use are critical, it is necessary the development of Application Specific Integrated Circuits (ASICs) that respond to the specific electronic interface challenges associated with CMOS compatible impedance sensor systems. Following this research line, Figure 1 shows the block diagram of a battery-operated impedance spectrometry multichannel micro-instrument based on the FRA technique. The actuation system is a high-resolution wide-range (from ~300 Hz to ~300 kHz) digitally programmable analog sinusoidal oscillator [13], which provides the stimulus to the variable impedance and the corresponding quadrature control signals to the read-out electronics. The read-out channel consists of a pre-conditioning amplifier followed by a dual-phase Lock-In Amplifier, which extracts the real and imaginary parts of the impedance [14]. Taking a further step to achieve a complete on-chip measurement solution, this work presents the design and characterization of a fully integrated low dropout regulator (LDO), which is the essential core block in the power management unit [15,16,17,18]. It must provide, from a 3.6 V LiPo battery—i.e., a short-lived source of energy delivering a decreasing voltage level as it discharges over time—a stable, noise-free, accurate and load-independent 1.8 V power supply voltage for the whole multichannel excitation and readout system.

Design guidelines are to optimize the size and especially the power consumption to satisfy these critical constraints of portable on chip devices, while keeping a suitable regulating performance for our application specifications: output voltage V_out_ = 1.8 V for battery-compatible input voltages V_BAT_ = 3.6 V–2.1 V, with a maximum load current of 50 mA over a 100 pF maximum capacitive load. Under these design terms, the LDO can supply up to 10 sensor signal-processing blocks, each block consisting of the respective excitation and readout systems. Besides, to obtain in the future a complete SoC measurement system, the whole design has been implemented in the same low cost CMOS process as [13,14], the UMC 0.18 μm 1P-6M CMOS technology, which provides transistors with 1.8 V–3.3 V nominal supplies, MIM (Metal-Insulator-Metal) capacitors (C_POX_ = 1.0 fF/μm^2^), and a high resistive polysilicon (HRP) layer (R_square_ = 1039 Ω/sq.).

Key design considerations for LDO regulators include stability, line/load regulation, line/load transient and power supply rejection (PSR). Conventional LDOs use an off-chip capacitor in the μF range at the output, both to guarantee stability and to minimize output voltage variations in the transient response. However, internal compensation is needed to attain a fully integrated solution that minimizes size and cost. Besides reliable on-chip compensation, operation with low quiescent current is mandatory to prolong the battery cycle. Nonetheless, a low quiescent current unavoidably slows the LDO transient responses, dominated by the slew-rate characteristic at the gate of the pass transistor. Finally, high precision regulation requires high loop gain, i.e., the use of high gain error amplifiers. Thus, for very low supply LDOs, a multi-stage error amplifier has to be employed. However, the compensation network of a fully integrated LDO with a multi-stage amplifier is not trivial, requiring advanced compensation techniques, such as damping factor control [19], Q-reduction [20] or enhanced multipath nested Miller [21]. Therefore, the challenge in CMOS LDO design is to achieve stability with reasonable on-chip compensation capacitance and minimum quiescent current while exhibiting good static regulating performance and fast transient behavior, since trade-offs between these parameters are interrelated.

In particular, with our design specifications, we have adopted a strategy that relies on using the simplest high-gain error amplifier, a telescopic structure which will be detailed next, to achieve good regulating performance and PSR while simplifying stability to the two-pole case, with a minimum-area minimum-quiescent-current solution, our two critical design requirements. To overcome the trade-off between power consumption (low quiescent current) and transient response, different techniques have been proposed, but they involve increasing the current and the circuit complexity of the resulting topology, thus degrading the power efficiency. For instance, the LDOs in [22,23] use current Miller amplification, i.e., a current amplifier in series with a capacitor that creates an auxiliary fast loop both to improve the transient response and to achieve internal frequency compensation. Adaptive techniques detect load variations through a relatively small current sensing transistor M_S_ in parallel with the power pass transistor. It generates a scaled copy of I_L_, that is next adequately injected directly at the gate of the pass transistor [24] or added to the bias current of the error amplifier, which is thus biased with a small fixed bias current plus an adaptive bias current proportional to I_L_ [25,26]. The associated circuit topology is simple, and thus compact. However, the transient improvement is only effective during transitions from low to high currents, but not for the opposite conversion, while the quiescent current becomes proportional to I_L_, increasing when the LDO is active. Alternatively, dynamic techniques rely on the employment of auxiliary current boosting paths to improve the transient behavior, which are only active during transient periods but that remain off in steady state. Therefore, the system can operate with reduced quiescent current, and then the charging/discharging current at the gate of the power transistor [27] or the biasing current of the error amplifier are increased momentarily [28,29,30].

Hence, the dynamic technique is the one exhibiting better current efficiency. That is why this paper applies this approach to achieve an internally compensated LDO regulator with enhanced time response thanks to the introduction of a novel dynamic current boosting bias circuit. Over other proposals based on this technique [27,28,29,30], this scheme manages to work with no additional quiescent current and minimal additional circuitry, thus resulting in an ultralow power LDO with very competitive static and dynamic regulating performances.

Some preliminary simulation results were presented in [31,32]. This paper is organized as follows: Section 2 describes the LDO regulator design. Section 3 reports the experimental characterization. Section 4 validates its application within the micro-instrument in Figure 1, designed for portable impedance measurement, and that includes a set of 10 SoC channels. Finally, in Section 5, conclusions are drawn.

## 2. Proposed LDO Design

**Figure 2:**
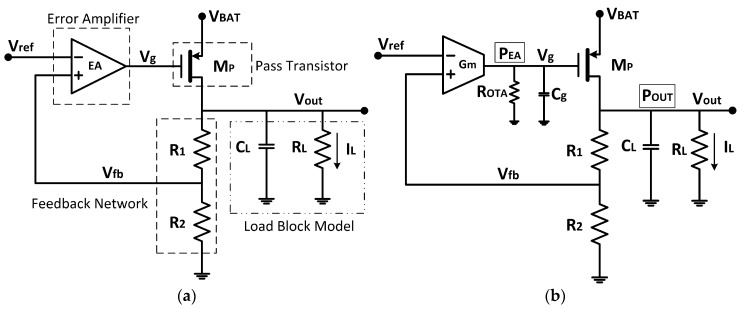
(**a**) Basic LDO voltage regulator topology; and (**b**) single stage OTA EA implementation.

Figure 2a shows the basic topology of a CMOS LDO regulator. It consists of an error amplifier (EA), a resistive feedback network and a PMOS transistor acting as the pass device between the input voltage V_BAT_ and the regulated output voltage V_out_ that powers the load, modeled through R_L_//C_L_. The feedback resistors R_1_–R_2_ sample variations on V_out_ due to variations on the input voltage and/or load current. This sampled voltage V_fb_ is compared to a voltage reference V_ref_, and the amplified difference continuously drives the pass transistor gate so that the output voltage is kept constant according to the relationship

(1) $$V_{\mathrm{out}}\approx \left(1+\frac{R_{1}}{R_{2}}\right)V_{\mathrm{ref}},$$

**Figure 3:**
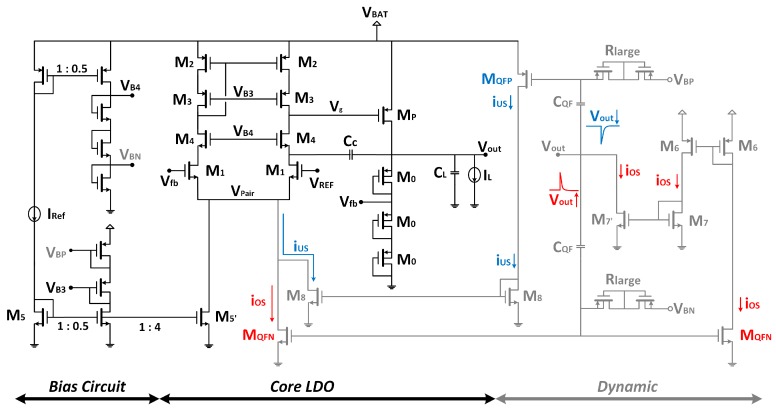
Schematic of the proposed CMOS LDO regulator.

Based on this architecture, the proposed fully integrated internally compensated LDO voltage regulator is shown in Figure 3. The voltage reference V_ref_ is an external 1.2 V reference.

### 2.1. LDO Core

With our design specifications (V_ref_ = 1.2 V; V_out_ = 1.8 V) from Equation (1) and assuming a static current of 4 μA flowing through resistances R_1_–R_2_ when I_L_ = 0 (I_fb_ = V_out_/(R_1_ + R_2_) = 4 μA), as a trade-off between low power consumption and moderate resistance values, it results *R*_1_ = 150 kΩ and R_2_ = 300 kΩ. They are implemented as active resistances using three identical PMOS transistors in diode configuration (M0, Figure 3) instead of as passive resistances to optimize area.

The size of the PMOS pass transistor is set to 9 mm/340 nm to guarantee operation in saturation, in the first order of approximation, for the maximum load current (50 mA) preserving a dropout voltage of V_do_ = V_DS,MP_ = 300 mV. Minimum transistor length (L = 0.34 μm for 3.3 V MOS transistors) is used to reduce the parasitic capacitance at the pass transistor gate: C_g_ ~12 pF (no load) and ~20 pF (maximum load).

The EA is a telescopic NMOS input differential pair Operational Transconductance Amplifier (OTA), which provides high gain—comparable to that of a two-stage topology—with the simplest single-stage OTA. In this way, high precision regulation can be achieved minimizing power consumption and relaxing the system stability. It drains a total current consumption of 2.5 μA (2 μA for the differential pair plus 0.5 μA to generate the cascode bias voltages V_B3_ and V_B4_ through diode-connected transistors). Its DC gain AEA is above 97 dB over the nominal battery supply operating range (2.1 V–3.6 V), and it renders a gain-bandwidth product GBW > 149 kHz with a phase margin PM = 89.6° considering a load capacitance equal to C_g_.

### 2.2. Stability

Since the LDO regulator structure is based on a negative feedback control loop to establish the constant output voltage, an important aspect is to ensure stability under all the operating conditions, that is, for all the voltage supply and load current ranges. Conventional LDOs add an off-chip capacitor in the order of ~μF at the regulator output that, besides settling the dominant pole, improves the transient response. These are called external compensated LDOs, because capacitances of such magnitude (μF) cannot be integrated within a reasonable area. Therefore, for SoC solutions, a different strategy must be used.

In our case, using as *EA* a single stage *OTA* (Figure 2b), the corresponding PMOS linear regulator is a second-order system. It presents a dominant pole associated to R_OTA_ and C_g_, where R_OiTA_ ≈ [(g_m2_r_o2_r_o3_||g_m4_r_o4_r_o1_)] is the OTA output resistance (parameters having their usual meaning) and C_g_ is the gate capacitance of the pass transistor,

(2) $$f_{P_{EA}}\approx \frac{1}{2\pi }\frac{1}{R_{\mathrm{OTA}}C_{g}},$$

The non-dominant pole is associated to the output LDO node and can be expressed as

(3) $$f_{P_{OUT}}=\frac{1}{2\pi }\frac{1}{R_{\mathrm{eq}}C_{L}}\approx \frac{1}{2\pi }\frac{I_{L}}{V_{\mathrm{out}}C_{L}},$$

where R_eq_ ≈ [(R_1_ + R_2_)||R_oP_||R_L_] ≈ R_L_ is the equivalent output load resistance, R_oP_ the output resistance of the pass transistor and C_L_ the load capacitance.

**Figure 4:**
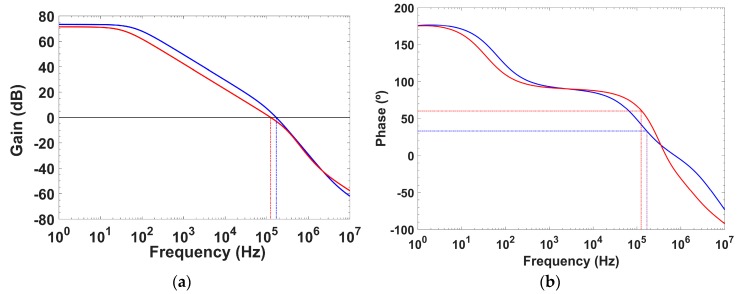
Simulated stability behavior with (red) and without (blue) cascode compensation for I_L_ = 0, V_BAT_ = 2.1 V: (**a**) gain; and (**b**) phase margin.

**Figure 5:**
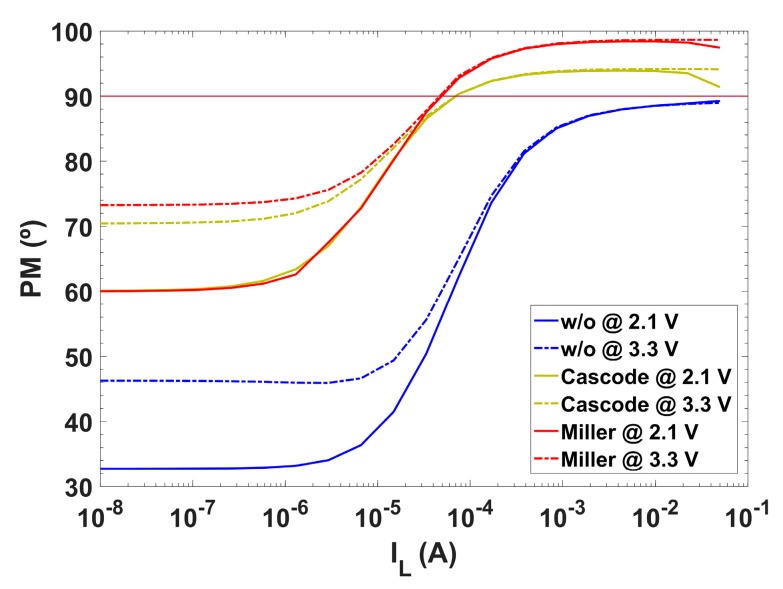
Simulated phase margin versus load current for: Cascode (C_c_ = 9.5 pF), Miller (C_c_ = 11 pF, R_c_ = 17.5 kΩ) and without compensation.

It is clear that, for high load currents, P_OUT_ increases moving towards higher frequencies and renders a stable system, but for low load currents P_OUT_ gets closer to the dominant pole, reducing the phase margin below the limit that guarantees stability. Thus, a cascode compensation technique, using a single C_c_ = 9.5 pF MIM capacitor (Figure 3) is adopted to accomplish pole splitting and stabilize the system, with the criteria of preserving a phase margin PM above 60° (Figure 4). This approach has been preferred over the classical Miller compensation technique, which requires a C_c_ = 11 pF, R_c_ = 17.5 kΩ network to attain the same 60° phase margin at I_L_ = 0; besides, the Miller solution exhibits for load currents I_L_ > 0.1 mA a further overcompensated phase margin response (Figure 5).

### 2.3. Transient Response

The combination of a low load capacitor in the LDO output node and the use of minimum quiescent currents to drive the large capacitor C_g_ at the gate of the power transistor overall results in voltage peaks and large settling times for the transient response. To improve this transient behavior without jeopardizing the quiescent current, a dynamic current bias boosting circuit (CBBC), shown in grey in Figure 3, is proposed. It consists of undershoot/overshoot (US/OS) detection circuits, with the corresponding US/OS driving circuits.

The undershoot (US) detection circuit is a quasi-floating gate PMOS transistor M_QFP_. Its gate voltage is tied to a DC biasing voltage V_BP_ through large resistive elements R_Large_—implemented using two series reverse biased PMOS diodes—and to the output node through a small valued MIM capacitor C_QF_ = 1 pF. In this way, under quiescent conditions, the M_QFP_ gate voltage takes the value V_BP_, which is fixed to a value V_SG_ = (V_BAT_–V_BP_) = 350 mV < |V_THP_|= 0.72 V that keeps M_QFP_ in the cut-off region. When the output voltage suddenly decreases, capacitor C_QF_ transfers the output voltage undershoot to the M_QFP_ gate, making V_SG_ > |V_THP_| and the transistor enters the on region. The generated current is copied through the current mirror M8, adding extra bias current to the error amplifier that speeds the discharge of capacitance C_g_. When V_out_ is approximately regulated back to its nominal value, M_QFP_ returns to the off region.

Similarly, the overshoot (OS) detection circuit is a quasi-floating gate NMOS transistor M_QFN_, with the gate voltage set to a DC biasing voltage V_GS_ = V_BN_ = 300 mV < V_THN_ = 0.59 V through R_Large_, and connected to the output node through C_QF_. In steady state, M_QFN_ is off, but when the output voltage suddenly increases, the overshoot will couple through C_QF_, triggering on the transistor. The generated current is added to the bias current of the EA, helping to charge the gate capacitance C_g_ and, as a result, V_g_ is increased to reduce I_L_. Besides, M_QFN_ is replicated and the current mirror M6-M7-M7’ sinks extra current at the output, helping to discharge the path formed by (R_1_ + R_2_) and C_L_.

Both V_BP_ and V_BN_ are generated from the same bias branches used to generate the cascode bias voltages to add no extra current. Therefore, the total quiescent current in steady state is only 7 μA (0.5 μA from the reference current I_ref_ + 4 μA from the feedback network +2 μA from the EA + 0.5 μA to generate all biasing voltages).

Compared to previous proposals based on the dynamic technique [27,28,29,30], the main advantage of the proposed current bias boosting circuit (CBBC) is that it effectively improves the transient response both with simpler circuitry (<4% of the total chip, including both C_QF_) and with no additional ground current, therefore not degrading the system power consumption and size. More in detail, the LDO in [27] makes use of a simple differential pair as error amplifier, with triple transient improved loops; it achieves similar regulating performances, exhibiting comparable FoMs, but with a quiescent current 3.6 times greater and twice the area. Output voltage spikes detection based on RC high pass filtering is implemented in [28]; however, this requires large capacitance and resistance values: the area of the HPF is more than half of the total chip area. The LDO in [29] uses a combination of a low power simple differential pair EA and two high-speed comparators to dynamically increase the bias current of the EA. However, to achieve a settling time of 200 ns, the comparators need 20.6 μA of the total 26 μA quiescent current, severely degrading the power consumption performance. A current-reused dynamic biasing circuit in the output of a two-stage EA using an NMOS-pass transistor to improve the load transient response with no extra current is implemented in [30]. However, this quiescent current is as high as 130 μA, and this solution needs a charge-pump voltage doubler driven by an external clock to bias this output stage allowing a drop-out voltage of 200 mV.

## 3. Experimental Validation

**Figure 6:**
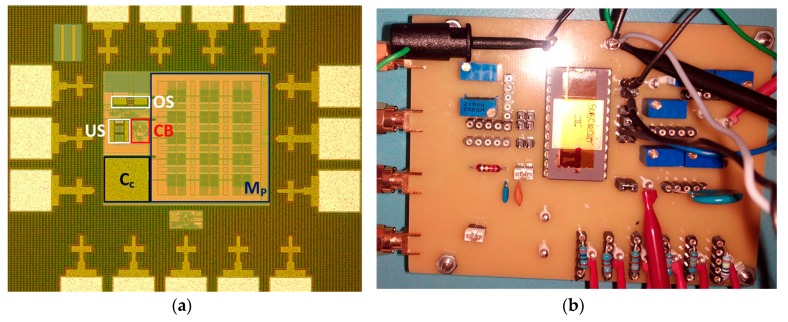
(**a**) Detail of the integrated LDO regulator (CB: Core (without M_P_) + Bias); and (**b**) LDO PCB test.

Figure 6a shows a microphotograph of the integrated LDO regulator. Its active area is 362 × 283 μm^2^, mostly occupied by the power PMOS transistor. A specific PCB was designed (Figure 6b) to complete its static, dynamic and high frequency (PSR) characterization.

### 3.1. Static Behavior

**Figure 7:**
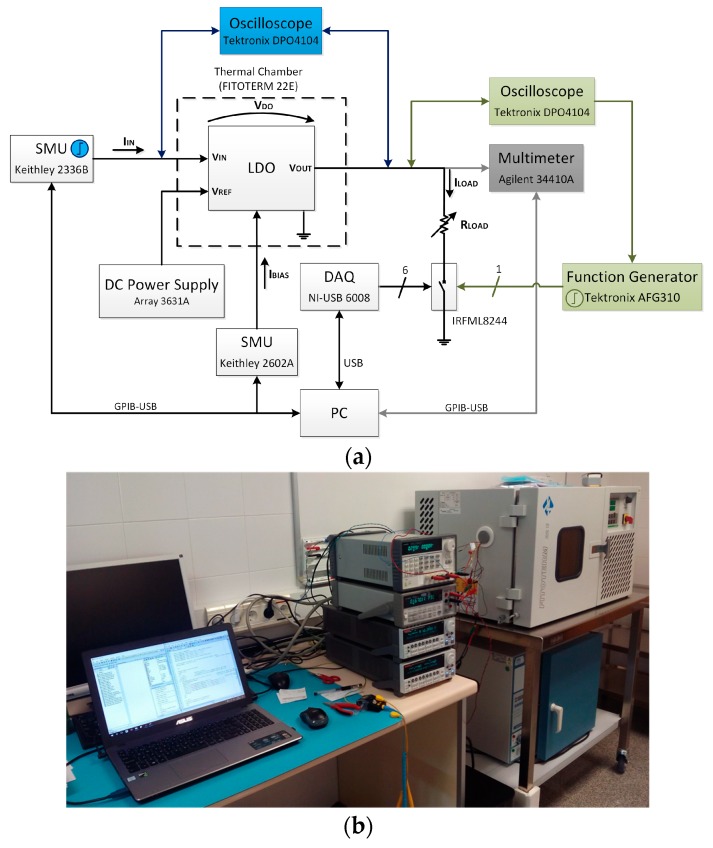
Measurement setup for the complete characterization of the LDO regulator: (**a**) block diagram of static (grey), transient load regulation (green) and transient line regulation (blue); and (**b**) experimental setup.

Figure 7 shows the measurement setup (Figure 7a shows the block diagram and Figure 7b shows a photograph of the experimental setup) for the characterization of the main static parameters: V_in_–V_out_ characteristic and drop-out voltage, quiescent current, line regulation LNR (circuit capacity to keep the specified output voltage in the range of input voltages) and load regulation LDR (circuit capacity to keep the specified output voltage under different load conditions). A DC Power Supply 3631A from Array (Array Electronic Headquarters, Nanjing, China) sets the 1.2 V reference voltage. To emulate different load currents (0, 1 μA, 10 μA, 100 μA, 1 mA, 10 mA and 50 mA) ,an array of six commuted resistances placed at the output of the LDO are used, each of them activated through a respective low impedance NMOS transistor IRFML8244 (R_DS(on),max_ = 41 mΩ) from IR (International Rectifier Headquarters, El Segundo, California, USA) acting as switches with their gates connected to the digital outputs of a Data Acquisition Card (DAQ) USB-6008 from NI (National Instruments Headquarters, Austin, TX, USA).

**Figure 8:**
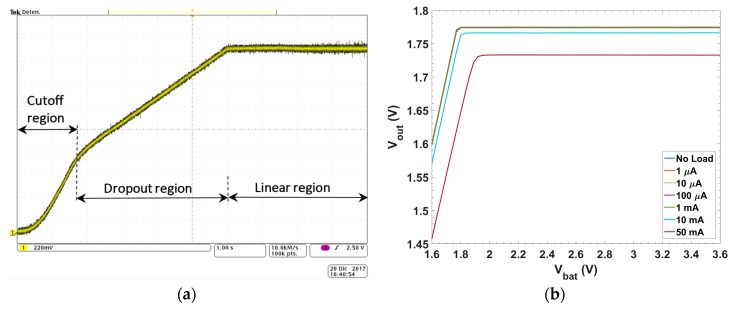

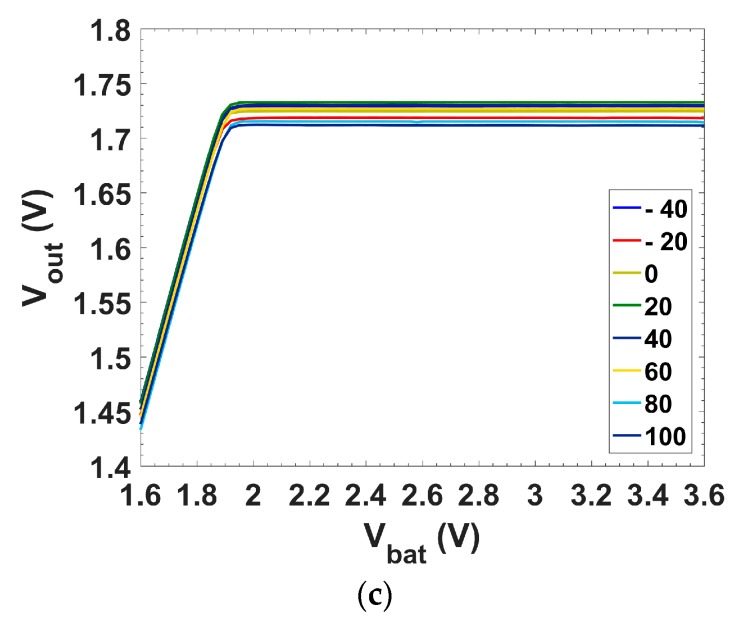
V_in_–V_out_ characteristic: (**a**) oscilloscope caption at I_L_ = 50 mA; (**b**) different current loads; and (**c**) different temperatures with maximum load current (50 mA).

Firstly, a DPO4104 Oscilloscope from Tektronix is used to corroborate the proper behavior of the integrated LDO. Figure 8a shows the static V_in_–V_out_ performance for I_L_ = 50 mA. Secondly, automatized measurements were accomplished to perform a complete V_in_–V_out_ characterization over different load currents. The input voltage V_BAT_ is provided by a Source Measure Unit 2336B (SMU) from Keithley (Keithley Instruments Headquarters, Cleveland, OH, USA) that allows, for each input V_BAT_, the simultaneous measurement of the quiescent current. The output voltage is measured with a Digital Multimeter of 6 1/2 digits 34410A from Agilent Technologies (Agilent Technologies Headquarters, Santa Clara, CA, USA). Tests have been performed in a range of temperatures that spans from −40 °C to 100 °C in 20 °C steps, using a thermal chamber FITOTERM 22E from Aralab (Aralab Headquarters, Sintra, Portugal). Figure 8b presents the obtained results, with a sweep of the supply voltage from 1.5 V to 3.6 V in 0.01 V steps, at room temperature (20 °C) for different load currents (from 0 to 50 mA). The LDO regulator provides a constant output voltage of 1.8 V for input voltages >1.94 V (V_do_ = 140 mV) with an error <4% for the worst case, corresponding to maximum load current. Next, in the range of −40 °C–100 °C in 20 °C steps, this same characteristic is measured for the most critical state, i.e., at maximum current. Results are shown in Figure 8c. The V_do_ remains over 140 mV and, in the linear region, the output voltage experiences a maximum variation of 20 mV over the 140 °C temperature range (~143 μV/°C).

**Figure 9:**
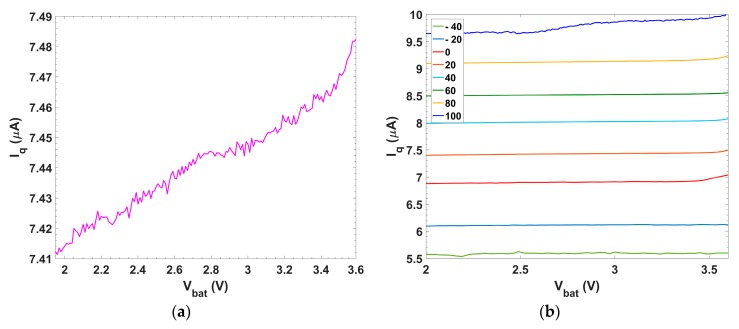
Quiescent current at: (**a**) room temperature; and (**b**) over different temperatures.

The measured quiescent current of the system over V_bat_ is shown in Figure 9a. Its average value is 7.45 μA, with a negligible difference (~70 nA) between the minimum and maximum battery voltage. Figure 9b shows the quiescent current against the battery voltage range for different temperatures. The value is kept constant at each temperature over the battery supply, increasing at a rate of ~32 nA/°C.

The Line Regulation (LNR) is the static variation at the output voltage ΔV_out_ due to a static variation of the input voltage ΔV_in_. It is typically specified by

(4) $$\mathrm{LNR}=\frac{\Delta V_{\mathrm{out}}}{\Delta V_{\mathrm{in}}}\;\left(\mathrm{mV}/V\right)=100\frac{\Delta V_{\mathrm{out}}}{\Delta V_{\mathrm{in}}}\frac{1}{V_{\mathrm{out}}}\;\left(\%/V\right),$$

**Figure 10:**
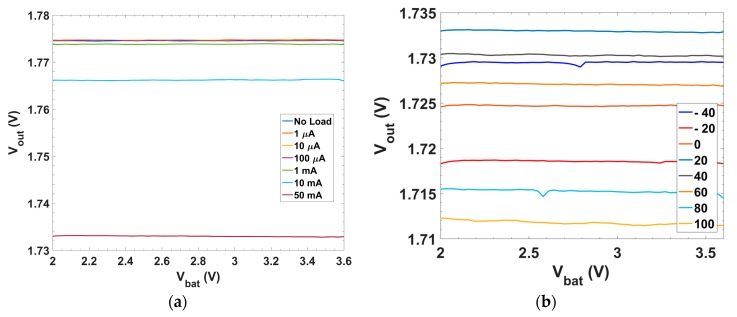
Line regulation characteristic, i.e., output voltage vs. input voltage: (**a**) for different load currents; and (**b**) for different temperatures under maximum load current condition.

Figure 10a presents the LNR performance (basically a zoomed version of Figure 7b in the LDO linear region). The variation of the output voltage through all the operating range for the worst case (I_L_ = 50 mA) provides a LNR = 0.081 mV/V. Figure 10b presents the LNR behavior over temperature. Load Regulation, defined as the static variation at the output voltage ΔV_out_ due to the static variation of the load current ΔI_L_, is typically specified by

(5) $$\mathrm{LDR}=\frac{\Delta V_{\mathrm{out}}}{\Delta I_{L}}\;\left(\mathrm{mV}/\mathrm{mA}\right)=100\frac{\Delta V_{\mathrm{out}}}{\Delta I_{L}}\frac{1}{V_{\mathrm{out}}}\;\left(\%/\mathrm{mA}\right),$$

**Figure 11:**
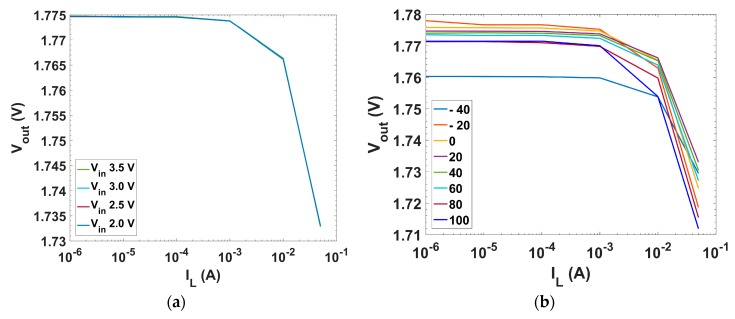
Load regulation characteristic, i.e., output voltage vs. load current: (**a**) for different input voltages; and (**b**) for different temperatures with 2.5 V input voltage.

Figure 11a presents the LDR for different input voltages within the operating range of the LDO regulator. The worst case (V_in_ = 2.0 V) provides a LDR = −0.82 mV/mA. Figure 11b presents the behavior over T.

### 3.2. Dynamic Behavior

**Figure 12:**
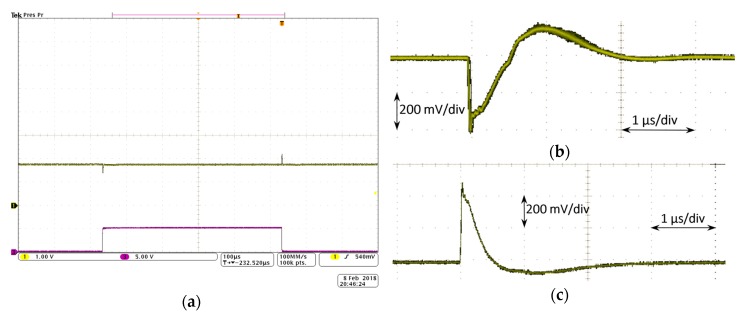
Load transient behavior: (**a**) with dynamic CBBC (green) output voltage and (purple) ON/OFF (50 mA/0 mA) of the switch that allows load current through; (**b**) US zoomed image; and (**c**) OS zoomed image.

The dynamic behavior of the LDO regulator is tested at room temperature (T ~ 20 °C). To characterize the transient load regulation (Figure 7, in green), the output voltage variation is measured for a current step from minimum to maximum load current, at a specific input voltage. That current step is obtained through an AFG310 Arbitrary Function Generator from Tektronix (Tektronix Headquarters, Beaverton, OR, USA) used to provide a square signal that opens and closes the NMOS transistor switch connecting the output voltage with a load current of 50 mA, switching in this way from 0 to 50 mA. The output voltage variation is captured with the oscilloscope. Figure 12 shows the oscilloscope screenshot for a current step from 0 to 50 mA (t_rise_ = 0.5 μs) with an input voltage of V_BAT_ = 3.6 V. The regulated output voltage with the dynamic CBBC (Figure 12a) shows an OS/US of ~480 mV/~400 mV with settling times of 2.5 μs/2.0 μs, respectively. Compared to the transient load regulation without the dynamic enhancement circuit, it shows an improvement of two orders of magnitude in the settling times and an important reduction on the OS/US voltage variations.

**Figure 13:**
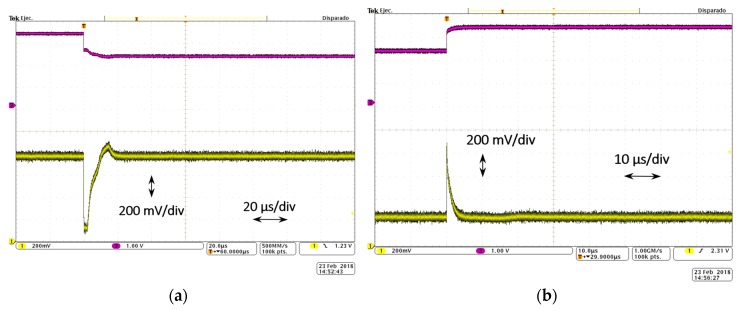
Line transient behavior: (**a**) undershoot response; and (**b**) overshoot response.

Characterization of the transient line regulation (Figure 7, in blue) sets an input voltage step within its linear range, at a specific load current. Figure 13 shows a screenshot of the transient line regulation for an input voltage step from 2.2 V to 3.2 V with a load current of 50 mA. The regulated output voltage shows an US (Figure 13a) of ~700 mV and a settling time of 20 μs, while the OS (Figure 13b) presents a voltage variation of ~600 mV and settling time of 4 μs.

### 3.3. Power Supply Rejection (PSR)

Finally, the PSR measures the capacity of the LDO regulator to reject ripple, of various frequencies, injected at its input [33]

(6) $$PSR=\left|20\log_{10}\left(\frac{V_{\mathrm{out}}}{V_{\mathrm{in}}}\right)\right|,\;\left(\mathrm{dB}\right)$$

**Figure 14:**
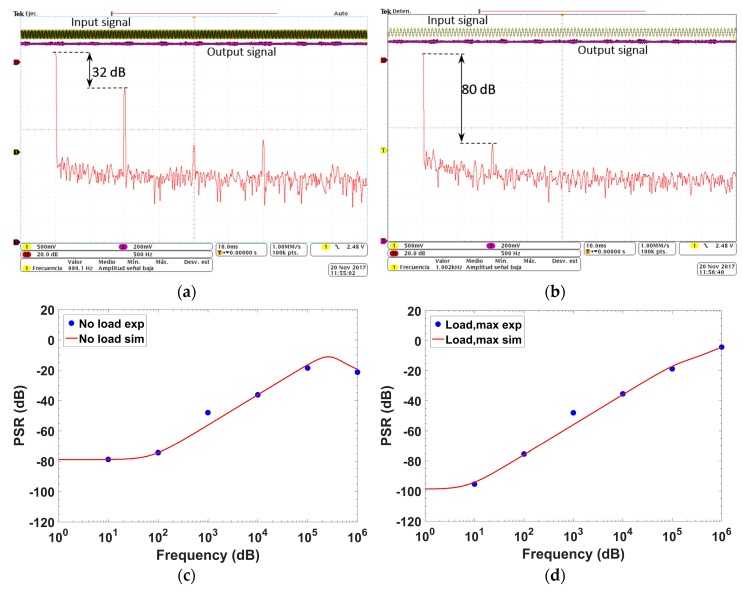
Oscilloscope screenshot for *PSR* calculation: (**a**) FFT of the input signal; (**b**) FFT of the output signal. *PSR* over frequency for: (**c**) minimum load; and (**d**) maximum load.

Figure 14 shows the *PSR* value at no load condition for an input signal of 0.1 V amplitude and 1 kHz frequency over a supply voltage of 2.8 V (green) and an output voltage centered at 1.8 V (purple). The FFT (red) shown in Figure 14a corresponds to the input signal (green) and the one in Figure 14b to the output signal (purple). A drop of ~48 dB in the 1 kHz signal is measured. Figure 14c shows the *PSR* for different frequencies for no load and maximum load current.

Note that, since the *PSR* largely depends on the feedback gain [34],

(7) $$A_{Fb}=\frac{A_{EA}}{\left(1+s/\omega_{P_{EA}}\right)}\frac{R_{2}}{R_{1}+R_{2}}$$

at low frequency; since *A_EA_* is high, the low frequency supply rejection is good as expected. However, beyond the frequency of the error amplifier pole *ω_P_EA__*, the feedback gain reduces and the *PSR* consequently degrades. The LDO is designed to work into a Lab-on-Chip micro-instrument directly powered by batteries, being the main interference expected from load transients (activation and deactivation of the different signal processing blocks supplied with the LDO regulator). Thus, the *PSR* has not been considered a critical design issue while more relevance is given to good DC regulation, fast transient response, ultra-low current and reduced area consumption.

Table 1 summarizes the main characteristics of the presented LDO regulator and compares the experimental results with other measured CMOS designs, with similar specifications. The proposed regulator attains within an area of 0.10 μm^2^ better overall line and load regulation with a reduction of the power consumption while it keeps similar time response parameters, operating for a range of temperatures from −40 °C to 100 °C. To better evaluate the performance of different designs, two figures-of-merit (FoM) are defined. The first one is expressed as

(8) $$FoM_{1}=\frac{C_{L}*\mathrm{LNR}*\mathrm{LDR}*I_{q}}{1000*I_{L,\max}}\;\left(s\right)$$

compares the regulation performance-power efficiency trade off, where C_L_ (pF), LNR (mV/V), LDR (mV/mA), I_q_ (μA) and I_L,max_ (mA) are the output capacitor, the line and load regulation, the quiescent current and the maximum load current. The factor 1000 is introduced to have FoM_1_ dimensioned in (s).

The second figure-of-merit is a widely adopted *FoM* [21,35] to evaluate the transient performance:

(9) $$FoM_{2}=\frac{T_{settle}*I_{q}}{I_{L,max}}\;\left(s\right)$$

In both cases, the smaller is the *FOM* value, the better is the performance metric. Besides, an α correction factor as proposed in [29] is introduced in both *FoMs* (*FOM_i_*^†^ in Table 1)

(10) $$\alpha =\frac{I_{q}+I_{L,min}}{I_{q}}$$

to take into account the minimum load current at which the LDO must operate, thus including the I_L,min_ requirement into I_q_. In this way FoM_1_**^†^** properly evaluates the regulation performance with the effective power consumption and FoM_2_**^†^** the transient response for a full load transition. According to Table 1, the proposed LDO achieves really competitive FoMs, rendering the best regulating–transient performance trade-off over a wide temperature range.

## 4. Micro-Instrument Application

**Figure 15:**
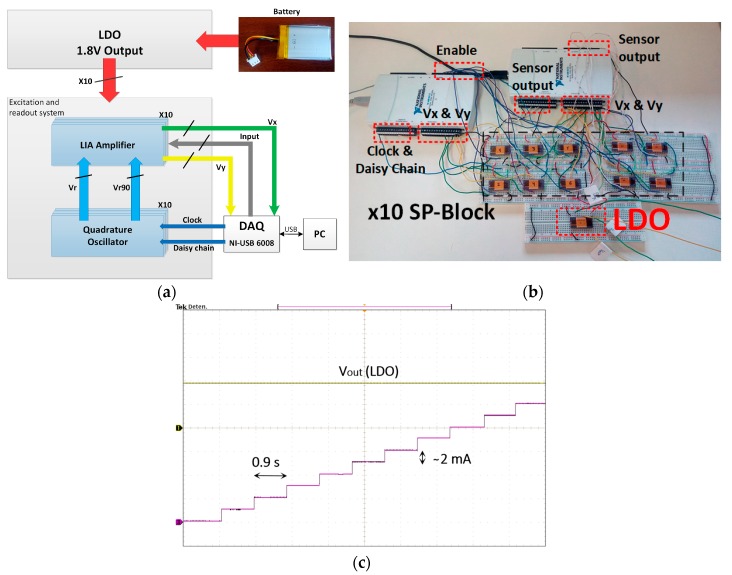
Multichannel LIA-based battery supplied micro-instrument: (**a**) Implementation; (**b**) block diagram; and (**c**) oscilloscope screenshot for the application of (green) LDO output voltage and (purple) activation of each signal-processing block.

To show the functionality of the proposed LDO regulator, the micro-instrument shown in Figure 1 was emulated, as shown in Figure 15a. The setup (Figure 15b) includes 10 CMOS lock-in based signal-processing (SP) blocks, each of them encapsulated in a 24-pin dual in line (DIL-24) package, consisting of one quadrature signal generator and a dual readout system. All of them are biased to 1.8 V using the proposed LDO regulator, encapsulated in a separate DIL-24 package (Figure 15a, down). Two DAQ USB-6212 from National Instruments emulate the impedance sensor signals and recover the corresponding output signals (Vx and Vy) provided by the 10 dual-channel LIAs. They also provide the clock signal required to configure the 12-bit registers that set the oscillators frequencies, sent in daisy chain.

Each individual processing IC presents a current consumption of ~2 mA, and can be individually activated and deactivated to verify the dynamic LDO output voltage behavior. Figure 15c shows the results achieved, when V_bat_ = 2.1 V, for the sequential activation (every 0.9 s) of the 10 circuits in the array, up to a total current consumption of ~20 mA. The proposed LDO regulator is perfectly capable of providing the demanded current while keeping a stable supply voltage, validating the suitability of the proposal for the target application.

## 5. Conclusions

This paper has presented an output capacitorless Low Dropout Regulator integrated in a 1.8 V–0.18 μm CMOS technology, capable of supplying a constant 1.8 V voltage to bias the excitation and readout channels of an array of sensors. The LDO regulator was specifically designed to meet the critical requirement conditions of battery-operated micro-instruments, such as low area (<0.10 mm^2^) and low power consumption (7.45 μm constant quiescent current, with 99.985% current efficiency), achieving remarkable state-of-art FoMs for the regulating–transient performance. This paves the way towards the achievement of miniaturized multichannel IS systems to the scale necessary for hand-held and point-of-care applications.

## Figures and Tables

**Table 1:** Comparison of CMOS Capacitorless LDO regulators.

Parameter	This Work	[18] 2012	[22] 2007	[25] 2012	[26] 2016	[27] 2011	[29] 2016	[30] 2018	[36] 2015
CMOS Tech. (μm)	0.18	0.35	0.35	0.35	0.18	0.35	0.35	0.18	0.065
V_in_ (V)	1.94–3.6	2.0–2.4	3	2.5–4	1.5–1.8	1.642–5	3.7	1.6–1.8	1.2
V_out_ (V)	1.8	1.073	2.8	2.35	1.2	1.5	3.25	1.4–1.6	1
V_do_ (mV) @ I_L,max_ (mA)	140 @ 50	47 @ 0.5	200 @ 50	150 @ 100	300 @ 50	142 @ 100	300 @ 50	200 @ 50	150 @ 10
I_q_ (μA)	7.45	35.7	65	7–17	2.4–242	27	26	130	50–90
C_Load_ (pF)	100	30	100	100	100	100	100	50	140
Line Regulation (mV/V)	0.081	39	~23	1	12.3	1.046	-	0.857	37.1
Load Regulation (mV/mA)	−0.82	13	~0.56	0.08	0.14	0.0752	~2.86	0.248	1.1
Full load ST (μs)	<2.5	-	15	~0.15 ^(a)^	~1.6	1	0.2 ^(b)^	0.04 ^(c)^	0.00115
*PSR* (dB) @ 1 kHz	‒48	‒38.1 @	‒57	-	<‒33 @	−60.6	~‒40	‒70	<‒21
		10 MHz			1 MHz				
Temp. range (°C)	‒140	37	-	-	-	-	-	-	-
Area (mm^2^)	0.10	~1	0.29	0.064	0.03	0.2	0.098		0.023
FOM_1_ (fs)	0.989	1.086 × 10^6^	1674.4	0.56–1.36	8.27–833.45	2.123	-		28567–51421
FOM_1_^†^ (fs)	0.989	-	1674.4	4.56–11.07	8.27–833.45	2.123	-		28567–51421
FOM_2_ (ns)	0.37	-	19.5	0.011–0.026	0.077–7.74	0.27	0.104		0.00575–0.01
FOM_2_^†^ (ns)	0.37	-	19.5	0.086–0.21	0.077–7.74	0.27	-		0.00575–0.01
FOM_1_^†^ xFOM_2_^†^ (ps)^2^	0.366	-	32650.8	0.392–2.325	0.64–6451	0.573	-		164.26–514.21

^(a)^ I_L_: 50 μA—max; ^(b)^ I_L_: 0.1 mA—max; ^(c)^ I_L_: 9 mA–40 mA; ^(†)^ FoM_1,2_ with the α factor applied.
